# Clinical Methods

**Published:** 1898-09

**Authors:** 


**Clinical Methods.** By Robert Hutchison, M.D., and Harry Rainy. Pp. xii., 552. London: Cassell and Company, Limited. 1897.

There are now many clinical manuals before the profession, but we think there is room for this one. It differs from most of the other manuals in that it covers the whole ground for the clinical examination of the patient, giving full directions as to case-taking, physical examination, investigation of the secretions, of pathological fluids, and some account of clinical bacteriology. We gather that the authors have endeavoured to write a book which shall form a complete clinical handbook and work of reference at the bedside and in the ward during the taking of a case. Nowadays, to accomplish this purpose in anything like a reasonable space, brevity must be everywhere studied and care taken to exclude all extraneous or impertinent matter.

This book well fulfils the purpose for which it was written, and we cordially recommend it. So far as our observation goes, it is accurate and trustworthy, and a vast amount of information is contained in a volume of moderate size. Of course, when detailed information is required on special points it must be supplemented by reference to larger works dealing with special branches only of clinical investigation.

The volume is well printed, badly bound, conveniently arranged for reference, has a good index, and the illustrations are numerous and excellent. There are also eight coloured plates. This is just the book that a clinical clerk should carry in his pocket, and the practitioner will find it equally convenient to have at hand for reference.

